# Rumen-protected lysine and methionine failed to improve the performance of late-nursing Awassi ewes regardless of dietary protein contents

**DOI:** 10.5455/javar.2024.k821

**Published:** 2024-09-29

**Authors:** Mofleh S. Awawdeh

**Affiliations:** Department of Veterinary Pathology and Public Health, Faculty of Veterinary Medicine, Jordan University of Science and Technology, Irbid, Jordan

**Keywords:** Amino acids, lysine, methionine, nitrogen, protein, sheep

## Abstract

**Objective:**

The current study investigated the effects of supplying ruminally protected amino acids (AA) (lysine, L; and methionine, M) and dietary protein levels on the performance of late-nursing ewes.

**Materials and Methods:**

Thirty-one Awassi ewes nursing single lambs were individually housed and assigned randomly to one of four treatment groups (2 × 2 factorial design). Ewes in treatment groups were (with supplemental RPL and RPM) or were not (without supplemental RPL and RPM) supplemented with lysine (8.5 gm/day) and methionine (4 gm/day) and were fed diets containing either 13.2 (moderate protein) or 11.1% (low protein) protein.

**Results:**

No interactions between supplemental AA and dietary protein levels were observed. Supplying ewes with L and M did not affect (*p* ≥ 0.06) their nutrient intake or their final body weights (BWs). Additionally, milk composition, yield, and efficiency were not affected by supplemental L and M. Decreasing dietary protein levels did not affect (*p* = 0.13) the final BWs, milk yield, composition, and efficiency but decreased (*p *< 0.01) nutrient intake of ewes.

**Conclusion:**

Under our study conditions, reducing the protein contents of the diets from 13.2% to 11.1% had no negative impact on late-nursing ewes. Regardless of dietary protein level, the beneficial effect of supplying L and M was not evident.

## Introduction

Decreasing dietary protein contents without negatively impacting ruminants’ performance has been a challenging target for ruminant nutritionists. Such a target provides several benefits to farmers (less production cost), the environment (less nitrogen excretion), and animals (less energy expenditure and better health). The concept of amino acid (AA) balancing with using rumen-protected (RP) AAs has been extensively investigated in dairy cows [1–3]. The most commonly used AAs are RP lysine (RPL) and RP methionine (RPM) because L and M were the common first and/or second-limiting AA in dairy diets [1]. Supplemental RPL and/or RPM showed variable improvements in milk yield and composition of dairy ewes [4–6], goats [7,8], and cows [9,10].

Although the AA-barrel principle and the first-limiting AA concept have been challenged in the latest dairy NRC publication [11], precise AA requirements for sheep and goats have not been established [12]. Additionally, published data on RPL and RPM is scarce in sheep [13] explicitly with low-protein diets [5].

Applying AA balancing allowed a reduction in dietary protein contents of dairy cows without negative effects on milk production [14]. Similarly, milk production of dairy cows fed diets insufficient in protein supply did not decrease when cows were supplied with RPL and RPM [15]. Improvements in milk yield and composition were observed when Comisana ewes fed low-protein diets were supplied with RPL and RPM [16].

Recently, we demonstrated no negative effects on performance when dietary protein level was decreased (from 17.0% to 15.1%) in early-nursing Awassi ewes [5]. Interestingly, RPL and RPM increased milk production in ewes fed the lower-protein diets [5]. With supplemental RPL and RPM (WLM), in the present study, we hypothesize that it is possible to decrease protein contents of diets fed to late-nursing ewes without detrimental effects on their performance. Our objective was to study the effects of decreasing dietary protein levels and supplying ruminally protected lysine and methionine on the performance of late-nursing ewes.

## Materials and methods

### Ethical approval

Procedures used in the study were approved by the Institutional Animal Care and Use Committee at Jordan University of Science and Technology (JUST #16/3/2/680). The study was carried out at JUST (Agricultural Research and Training Unit-ARTU).

### Animals and design

Awassi ewes [*n =* 31; initial body weight (BW) = 56.0 kg ± 7.5 SD] were enrolled in the current study. Ewes were in their late-nursing stage (average 6 weeks of lambing) and were individually housed (pen of 1.5 × 0.75 m) with their lambs. Pens were assigned randomly to one of four (2 × 2 factorial design) treatment groups: with (4 gm/day/ewe of RPM plus 8.5 gm/day/ewe of RPL; WLM) or without supplemental RPL and RPM (WOLM) and two levels of dietary protein [13.2% or 11.1%; moderate protein (MP) or low protein (LP)]. In the randomization of treatments, milk yield and BW of ewes recorded at the start of the study were considered.

Treatment diets were isocaloric and were formulated to meet ewes’ nutrient requirements [17]. Diets (total-mixed rations) were offered to ewes at 0800 and 1500 hours. Supplemental RPL and RPM were dressed on top of the morning portion. Each gram of RPL and RPM provided, respectively, 0.20 and 0.25 gm/ewe/day of metabolizable L and M. Offered and refused feeds for each pen were recorded daily, and the offered feed was adjusted to ensure *ad libitum* consumption. Clean water was available to ewes all the time.

The study lasted for 4 weeks. Before the morning feeding, the BWs of ewes were measured weekly. To estimate milk yield, ewes were hand-milked using two-oxytocin (10 IU) injections as detailed by Fernandez et al. [18] with some changes. Briefly, the two oxytocin injections were separated at a 3-h interval and ewes were hand-milked after each injection to measure milk yield during the 3-h. Lambs were separated from their dams between the oxytocin injections (3 h). Milk samples were collected (125 ml) after the second oxytocin injection and sent immediately for analysis.

### Samples collection and analytical methods

Refused feeds were recorded for each pen and sampled daily. Daily refused feed samples were composited after the study and analyzed [19] for dry matter (DM), ether extract (EE), organic matter (OM), and nitrogen (N). Samples were also analyzed for acid detergent fiber (ADF) and neutral detergent fiber (NDF) (ANKOM Technology Corporation, Fairport, NY). Weekly milk samples were analyzed for fat, lactose, protein, and solid nonfat (Milkoscope, Julie C8 automatic, Germany).

### Statistical analysis

The SAS System [20] was used for analysis. The statistical model for BWs of ewes contained the effects of RPL and RPM, dietary protein levels, and the interactions among them.

The model for repeated measures (DMI, milk yield, and milk composition) contained the effects of supplemental RPL and RPM, dietary protein levels, time (week), and the interactions among them. Per analysis, the appropriate covariance structure (autoregressive order one, compound symmetry, unstructured, or combination of autoregressive order one and structured) was selected. For milk yield analysis, data for the first week was used as a covariate.

The LSMEANS was used to estimate treatment means. Least squares means were separated and compared using the *t*-test. A *p*-value below 0.05 was considered significant.

## Results

### Treatment diets

Diets were, as intended, isocaloric [average 9.55 MJ/kg of metabolizable energy (ME)] and contained comparable nutrients except for crude protein (CP), L, and M contents (Table 1). As planned, the LP diet contained lower CP (11.1% *vs.* 13.2%), L (0.43% *vs.* 0.56%), and M (0.16% *vs.* 0.18%) than the MP diet.

### Ewes performance

The final BW of ewes did not change (*p* ≥ 0.75) by dietary treatments (Table 2). All ewes lost weight at the conclusion of the study. However, ewes that were supplied with RPL and RPM lost less (*p* < 0.01) weight (3.4 *vs.* 6.7 kg, respectively, for the WLM *vs.* WOLM group), particularly for the MP group.

The effects of RPL and RPM on milk composition, milk yield, and nutrient intake of nursing ewes are presented in Table 3. For all data in Table 3, interaction effects (between treatments and week or between supplemental RPL and RPM and dietary protein levels) were not (*p* ≥ 0.15) statistically significant. Supplemental RPL and RPM did not affect (*p* ≥ 0.93) nutrient intake of ewes but increased (*p *< 0.01) L (12.7 *vs*. 11.0 gm/day) and M (4.8 *vs.* 3.8 gm/day) intake. Ewes fed the LP diets (LPWOLM and LPWLM) had lower (*p* < 0.01) DMI and subsequently lower intakes of OM, CP (243 *vs.* 296 gm/day), EE, L (10.3 *vs.* 13.4 gm/day), M (4.0 *vs.* 4.6 gm/day), and ME. Although ewes fed the LP diets (LPWOLM and LPWLM) had lower DMI compared to the MP diets (MPWOLM and MPWLM), they had similar NDF and ADF intakes as a result of higher NDF (37.6% *vs.* 36.4%) and ADF (16.0% *vs.* 15.5%) contents in the LP compared to the MP diets.

**Table 1.** Ingredient and chemical composition of dietary treatments.

Item	Dietary treatment^1^	
	MP	LP
Ingredient (% DM)		
Barley	58.7	63.2
Wheat hay	26.9	27.9
Soybean meal	11.5	6.0
Limestone	1.7	1.7
Salt	1.1	1.1
Vitamin/minerals premix^2^	0.1	0.1
Nutrient (% DM)		
DM	90.3	90.2
OM	90.2	90.1
CP	13.2	11.1
NDF	36.4	37.6
ADF	15.5	16.0
EE	1.2	1.2
Lysine^3^	0.56	0.43
Methionine^3^	0.18	0.16
Metabolizable energy (MJ/kg DM)	9.6	9.5

^1^MP = moderate protein and LP = low protein. DM, dry matter; OM, organic matter; CP, crude protein; NDF, neutral detergent fiber; ADF, acid detergent fiber; EE, ether extract.

^2^Composition per 1 kg contained (vitamin A, 2,000,000 IU; vitamin D3, 50,000 IU; vitamin E, 500 mg; vitamin C, 1,000 mg; vitamin K_3_, 20 mg; Ca, 200 gm, P, 80 gm; Mg, 40 gm; Fe, 500 mg; Zn, 2000 mg; Mg. 1,000 mg; Cu, 300 mg; Se, 100 mg; I, 80 mg; Co, 50 mg).

^3^Based on tabular values (NRC, 2007).

Supplying ewes with RPL and RPM did not significantly (*p* ≥ 0.06) affect their milk efficiency, yield, or composition. There was a tendency (*p* = 0.08) for milk efficiency to be lower for ewes supplemented with RPL and RPM. Decreasing dietary protein did not significantly (*p* ≥ 0.13) affect milk yield, composition, and efficiency.

## Discussion

Because data about using RPL and RPM in sheep is limited in the literature [5,13], it was not possible to directly compare the current results with published data.

### Treatment diets

The protein content of our MP diet was within the lower limit of diets that are commonly (14.0% to 18.0% CP) fed to nursing Awassi ewe [21,22] or other breeds [16,23]. Our LP diet is comparable to the NRC [17] recommendations but rarely fed to nursing Awassi ewes in Jordan or other countries in the region.

### Ewes performance

Although ewes lost weight in all treatment groups, ewes supplied with RPL and RPM lost less weight compared to the unsupplemented group. Inconsistent with our results, BW change of lactating [16] or nursing [23] ewes were not affected by RPL and RPM when ewes did not lose weight. Similarly, RPL and RPM supplementation did not affect the BW change of ewes when Awassi ewes lost a slight (<2 kg) BW during the early lactation period [5]. This suggests a beneficial effect (by reducing the magnitude of loss) of RPL and RPM for ewes that are likely to lose substantial weight during the lactation period.

**Table 2.** Effects of supplemental lysine and methionine on BW of late-nursing ewes feed two levels of dietary protein.

Dietary treatment^1^	MP		LP			*p-*value^2^		
Item	WOLM	WLM	WOLM	WLM	SEM	CP*LM	CP	LM
*n*	8	8	7	8				
Initial BW, kg	59.1	53.9	55.7	54.4	2.66	0.48	0.62	0.25
Final BW, kg	51.3	51.2	50.1	50.8	2.40	0.97	0.75	0.90
BW change, kg^3^	−7.8^a^	−2.7^b^	−5.6^a,b^	−4.2^b^	1.16	0.13	0.80	<0.01

^a, b^Within row, means with different superscript are different at *p* value < 0.05.

^1^Dietary treatments were arranged according to the 2 × 2 factorial design with or without supplemental RPL and RPM (0 or 8.5 plus 4 gm/day/ewe of RPL and RPM; WOLM or WLM) and two levels of dietary protein (13.2% or 11.1%; MP or LP).

^2^P-values for effects of dietary protein (CP), supplemental lysine and methionine (LM), and interaction (CP*LM).

^3^Calculated as BW change = final BW – initial BW.

**Table 3.** Effects of supplemental lysine and methionine on nutrient intake, milk yield, and milk composition of late-nursing ewes feed two levels of dietary protein.

Dietary treatment^1^	MP		LP			*p-value* ^2^		
Item	WOLM	WLM	WOLM	WLM	SEM	CP*LM	CP	LM
*n*	8	8	7	8				
Nutrient intake, gm/day								
DM	2,236^a^	2,254^a^	2,204^b^	2,183^b^	13.6	0.16	<0.01	0.94
OM	2,017^a^	2,033^a^	1,985^b^	1,967^b^	13.1	0.16	<0.01	0.94
CP	295^a^	298^a^	245^b^	242^b^	1.7	0.16	<0.01	0.96
NDF	816	823	829	821	5.1	0.17	0.32	0.93
ADF	346	350	353	349	2.3	0.15	0.19	0.96
EE	27^a^	27^a^	26^b^	26^a^	0.2	0.18	<0.01	0.96
Lysine	12.5^b^	14.4^a^	9.5^d^	11.1^c^	0.07	0.19	<0.01	<0.01
Methionine	4.0^c^	5.1^a^	3.5^d^	4.5^b^	0.02	0.18	<0.01	<0.01
ME, MJ/d	21.5^a^	21.6^a^	20.9^b^	20.7^b^	0.13	0.15	<0.01	0.95
Milk, gm/d	763	664	730	722	35.2	0.22	0.73	0.16
Milk efficiency (kg milk/kg DMI*100%)	33.6	28.3	36.7	32.0	2.8	0.91	0.24	0.08
Milk composition, %								
Solid nonfat	11.6	11.6	11.9	11.8	0.14	0.72	0.13	0.94
Fat	9.4	9.9	9.1	9.5	0.36	0.92	0.36	0.20
Protein	4.3	4.3	4.3	4.4	0.05	0.32	0.45	0.94
Lactose	6.3	6.4	6.3	6.4	0.04	0.76	0.74	0.06
Composition yield, gm/d^3^								
Solid nonfat	88	74	96	82	7.5	0.97	0.29	0.08
Fat	70	63	73	66	6.9	0.99	0.66	0.36
Protein	33	27	35	31	2.8	0.80	0.35	0.11
Lactose	48	40	51	45	4.2	0.90	0.39	0.13

^a, b, c, d^Within row, means with different superscript are different at *p* value < 0.05.

^1^ Dietary treatment were arranged according to the 2 × 2 factorial design with or without supplemental RPL and RPM (0 or 8.5 plus 4 gm/day/ewe of RPL and RPM; WOLM or WLM) and two levels of dietary protein (13.2% or 11.1%; MP or LP).

^2^*p*-values for effects of dietary protein (CP), supplemental lysine and methionine (LM), and interaction (CP*LM).

^3^Calculated as composition yield = composition percentage*milk yield.

Decreasing dietary protein contents did not affect the final BW or BW change of our ewes. Consistent with our results, BW of lactating [16] or nursing [23] ewes did not change with decreasing protein content of the diets, respectively, from 15.7% to 12.9% or from 16.3% to 10.2%. Recently, reducing dietary protein from 17.0% to 15.1% did not affect the BW change of early-nursing Awassi ewes [5].

Supplying our ewes with RPL and RPM did not affect DMI, milk composition, milk yield, or milk efficiency. Consistent with our results, supplying RPL and RPM did not improve the milk yield of black-faced ewes irrespective of dietary protein contents (16.3% or 10.2%; [23]). On the contrary, RPL and RPM increased the milk yield of Comisana ewes at both (15.7% or 12.9%) protein levels in the diet [16].

Recently, supplying RPL and RPM did not affect the DMI or milk composition of early-nursing Awassi ewes [5] regardless of dietary protein contents. Awawdeh [5] observed an interaction effect between supplemental RPL/RPM and dietary protein contents on milk yield and efficiency. The highest efficiency (due to higher milk amount) was observed for ewes fed the lower protein diet (15.1% *vs.* 17.0%) and supplied with RPL and RPM [5]. In the current study, the interaction between supplemental RPL/RPM and dietary protein was not evident for milk yield and efficiency. This suggests that the interaction between supplemental RPL/RPM and dietary protein might depend on the dietary protein level (17.0% *vs.* 15.1%; [5] compared to 13.2% *vs.* 11.1%; the current study) and/or stage of lactation (early nursing; [5] *vs.* late nursing; the current study) that deserves further investigation.

In contrast to our results, DMI of ewes was not affected by a decrease (from 16.3% to 10.2%) in dietary protein [23]. Recently, decreasing dietary protein levels from 17.0% to 15.1% did not affect the DMI of Awassi ewes [5]. In the present study, decreasing protein contents of the diet reduced DM and CP intake of ewes. Decreased protein intake in the current study might have negatively impacted the ruminal ecosystem and nutrient digestibility (e.g., NDF and ADF) and, subsequently, the DMI of ewes [24].

Decreasing dietary protein (from 13.2% to 11.1%) had no effects on the milk composition or yield of our ewes. Likewise, the milk yield of black-faced ewes was not depressed with decreasing protein contents of the diet (from 16.3% to 10.2%) during the early-nursing period [23]. After weaning their lambs, the milk yield of Comisana ewes did not decrease with decreasing protein content from 15.7% to 12.9% in the diet [16]. Recently, milk production and composition of early-nursing Awassi ewes were not negatively impacted by reducing the protein contents of the diet from 17.0% to 15.1% [5].

Reducing dietary protein contents did not depress the milk production of our ewes. This suggests an adequate protein supply for ewes fed the LP diet or/and a greater contribution to milk protein synthesis from body protein stores [16,23]. Our ewes had about 140% and 120% of the suggested intake of metabolizable protein [17] for the MP and LP diets, respectively, based on the actual DMI and milk yield. Under similar conditions, the current study suggests that dietary protein can be lowered to 11.1% without negatively affecting the milk yield of late-nursing ewes. Our results go along with the global interest in reducing the protein contents of diets fed to ruminants without adversely affecting their performance [25,26].

## Conclusion

Regardless of dietary protein contents, RP lysine and methionine did not improve milk composition or the amount of late-nursing ewes. Although it depressed DMI, decreasing protein contents of the diets (from 13.2% to 11.1%) had no detrimental effects on milk composition or the amount of late-nursing ewes. Under the conditions of the current study, the protein contents of diets offered to late-nursing ewes can be reduced to 11.1% without a need for supplemental RPL and RPM. The reduced feed intake and weight loss of ewes observed in the current study deserve further investigation.

## List of Abbreviations

AA, amino acid; ADF, acid detergent fiber; ARTU, Agricultural Research and Training Unit; BW, body weight; CP, crude protein; DM, dry matter; DMI, dry matter intake; EE, ether extract; JUST, Jordan University of Science and Technology; L, lysine; LP, low protein; M, methionine; ME, metabolizable energy; MP, moderate protein; N, nitrogen; NDF, neutral detergent fiber, OM, organic matter; RP, rumen-protected; RPL, rumen-protected lysine; RPM, rumen-protected methionine; WLM, with supplemental RPL and RPM; WOLM, without supplemental RPL and RPM.
